# *Bartonella* spp. in Small Mammals and Their Fleas in Differently Structured Habitats From Germany

**DOI:** 10.3389/fvets.2020.625641

**Published:** 2021-01-18

**Authors:** Anna Obiegala, Martin Pfeffer, Daniel Kiefer, Matthias Kiefer, Nina Król, Cornelia Silaghi

**Affiliations:** ^1^Comparative Tropical Medicine and Parasitology, Ludwig-Maximilians-Universität München, Munich, Germany; ^2^Institute of Animal Hygiene and Veterinary Public Health, University of Leipzig, Leipzig, Germany; ^3^Bavarian State Collection of Zoology, Munich, Germany; ^4^Institute of Infectiology (IMED), Friedrich-Loeffler-Institut, Federal Research Institute for Animal Health, Greifswald, Germany

**Keywords:** *Apodemus flavicollis*, *Clethrionomys glareolus*, *Ctenophthalmus agyrtes*, *Megabothris turbidus*, *Bartonella grahamii*, Europe

## Abstract

Most *Bartonella* spp. are transmitted by fleas and harbored by small mammals which serve as reservoirs. However, little is known about the composition of fleas and their *Bartonella* spp. from small mammals in Central Europe. Therefore, the aims of this study were to investigate flea communities on small mammals from three differently structured sites (urban, sylvatic, renatured) in Germany as well as the prevalence of *Bartonella* spp. in small mammals and their parasitizing fleas. In total, 623 small mammals belonging to 10 different species (the majority were *Myodes glareolus* and *Apodemus flavicollis*) were available. Fleas were removed from the small mammals' fur, morphologically identified and DNA was extracted. To detect *Bartonella* spp., two conventional PCRs targeting the gltA gene and the 16S−23S rRNA intergenic spacer were carried out followed by sequencing. Obtained sequences were compared to those in GenBank. In total, 1,156 fleas were collected from 456 small mammals. Altogether, 12 different flea species (the majority were *Ctenophthalmus agyrtes, Nosopsyllus fasciatus*, and *Megabothris turbidus*) were detected. At the urban site mostly *Leptopsylla segnis* and *N. fasciatus* were collected which may be vectors of zoonotic pathogens to companion animals. The overall prevalence for *Bartonella* in small mammals was 43.3% and in fleas 49.1%. Five different *Bartonella* spp. were detected in small mammals namely *B. grahamii, B. taylorii, B. doshiae, Bartonella* sp. N40 and uncultured *Bartonella* sp. whereas in fleas four *Bartonella* spp. were found which were with the exception of *B. doshiae* identical to the *Bartonella* species detected in their small mammal hosts. While *B. grahamii* was the only zoonotic *Bartonella* sp. most *Bartonella* strains found in fleas and small mammals belonged to uncultured *Bartonella* spp. with unknown zoonotic potential. This study showed a high diversity of flea species on small mammals from Germany. Further, high prevalence rates of *Bartonella* species were detected both in fleas and in their mammalian hosts. Several different *Bartonella* species with a high genetic variability were discovered. Especially at the urban study sites, this may pose a risk for *Bartonella* transmission to companion animals and humans.

## Introduction

Bartonellosis, which can result in severe clinical symptoms in humans and their companion animals, is caused by the facultative intracellular alpha-proteobacteria *Bartonella* spp. (order Rhizobiales, family Bartonellaceae) (1). *Bartonella* spp. are arthropod-borne bacteria and mainly transmitted by fleas, lice, deer keds, and sandflies (2–5). Bartonellae are highly adapted to one specific or few closely related mammalian reservoir hosts in which they can cause long-lasting bacteremia. In contrast, infections in incidental hosts may evoke disease with a broad range of symptoms (6, 7). Amongst the most common reservoir hosts are cats, rodents and other small mammals. Phylogenetic analyses based on sequence data from *rpoB, gltA, ribC*, and *groEL* genes revealed four different deep-branching Eubartonellae lineages and additionally *Bartonella australis* (8). *Bartonella tamiae* and *Bartonella apis* could build two additional separate lineages, which is however not yet confirmed. Lineage 4 is the most diverse group regarding the variety of *Bartonella* spp. as well as reservoir host species. Thus far, the highest prevalence and highest diversity of *Bartonella* spp. were described in rodents. Five of these rodent-associated *Bartonella* spp. are known to be hazardous to human health (*Bartonella grahamii, Bartonella elizabethae, Bartonella vinsonii* subsp. *arupensis, Bartonella washoensis*, and *B. tamiae*) (9). In studies from Poland (10, 11), Sweden (12), France (13), and the UK (14) the prevalence of *Bartonella* spp. in rodents ranged from 0 to 72.2%. Fleas are suggested to serve as main vectors for *Bartonella* spp. which are associated with rodents (15, 16). Previous studies showed that fleas may transmit *Bartonella* spp. experimentally to their mammalian hosts (17, 18). Moreover, there are several epidemiologic studies based on the molecular analysis showing that rodent-associated fleas are also infected with *Bartonella* spp. in nature (19–21). Studies from the USA, Afghanistan, and Israel reported prevalences between 15.5 and 95% for *Bartonella* spp. in fleas collected from rodents and small mammals (19–22). The knowledge on the species diversity of fleas on small mammals and the *Bartonella* prevalence are very scarce in Central Europe. Small mammal species build the vast majority of hosts for over 50 different flea species (23). In Germany, there are only four reports about small mammal fleas from the last century and only one report which is more recent (19). Recently, our group reported high prevalences of *Bartonella* spp. in rodents (65.8%) and their associated fleas (54.1%) in Germany (24). Further studies on the prevalence and species diversity of *Bartonella* in rodents and especially their parasitizing flea species are scarce in Germany. The previous study by our group showed results from one location and the sample size examined did not allow statistical associations. Thus, the objectives of the present study were: (1) detection of flea species parasitizing small mammals and (2) detection of *Bartonella* spp. in small mammals and their fleas and (3) detection of associations between small mammals, fleas and *Bartonella* species.

## Materials and Methods

### Study Areas

To collect small mammals, traps were placed at three sites of urban, sylvatic or recultivated character. These locations were previously selected for field studies by our group (25, 26). The urban area (R1) “Dörnbergpark” (7.4 ha, 49°00′55.72″N, 12°05′08.89″E) is situated in the city centre of Regensburg, Bavaria, Southern Germany. It is a small well-tended park which was described in detail before (26, 27). The sylvatic area (T) “Angelberger Forst” (641 ha, 48°06′36.42″N, 10°34′33.40″E) is a large forest located in Bavaria, Southern Germany (28). The recultivated site (S) consisted of three trapping localizations (51°15′32.2″N, 12°21′02.5″E; 51°17′01.3″N, 12°21′00.6″E; 51°26′97.2″ N, 12°32′25.6″ E) which were previously examined by our group and named as sites “E,” “F,” and “G” (25). This area is surrounding a lake which was a former open pit brown coal mining region near Leipzig, Saxony, Eastern Germany (436 ha).

### Sampling of Small Mammals and Their Fleas

Altogether, 50 Sherman© live animal traps (H. B. Sherman Traps, Inc., Tallahassee, Fla., U.S.A.) were placed at each Bavarian site between July and October in 2012 and between April and September in 2013. In Saxony, 60 traps were placed between March and October in 2012 and between January and September in 2013 (official permit Site S: AZ 36.11–36.45.12/4/12-001, Site R1: 55.1-8646.4-140, Site T: 55.1-8646-2/30). Traps were placed for two consecutive nights per month and site and checked twice a day. Collected small mammals were anesthetized with CO_2_, then euthanized by cervical dislocation and stored at −80°C. Small mammals were morphologically identified using taxonomic keys (29). Additionally, randomly selected rodents (15 *Apodemus sylvaticus*, 14 *Myodes glareolus*, and 23 *A. flavicollis*, 5 *Microtus arvalis*, 1 *Mi. agrestis*) as well as all shrews (*Sorex* spp.; *n* = 5) (by-catch found dead in traps) and least weasels (*Mustela nivalis*; *n* = 2) were identified by conventional PCR targeting the cytochrome b gene (354 bp) (30). A complete necropsy was performed with the collection of spleen samples. Cross contamination may be ruled out during dissection as each small mammal was handled with its own set of dissection instruments. Disinfection of working surfaces was performed after each individual and gloves were changed. Fleas were collected with tweezers from the fur during small mammal dissection. Fleas were stored individually in 100 μl RNALater (Qiagen, Hilden Germany) until morphological identification under a stereomicroscope (31, 32). Detailed information about trapping procedures and sampling sites have been given before (25–28).

### Small Mammal Samples Made Available From Previous Studies

In a previous study by our group, 623 small mammals belonging to 10 different species (395 *My. glareolus*, 172 *A. flavicollis*, 6 *A. agrarius*, 35 *A. sylvaticus*, 6 *Mi. arvalis*, 1 *Mi. agrestis*, 2 *M. nivalis*, 4 *Sorex coronatus*, one *Sorex araneus*, and one *Talpa europaea*) were captured (26, 33).

### DNA Extraction

DNA was extracted from all collected rodents' spleens and from a preselected number of fleas (*n* = 450) which were collected from the small mammals. The DNA extraction was carried out for each sample individually with the QIAamp DNA Mini Kit (Qiagen, Hilden, Germany) as previously described (20). The quality and quantity of the extracted DNA samples were measured spectrophotometrically (NanoDrop ND-1000, Erlangen, Germany). DNA samples exceeding a concentration of 40 ng/μl were additionally diluted with elution buffer in order to avoid false negative results.

### Polymerase Chain Reaction and Sequence Analysis

DNA samples were tested for the presence of *Bartonella* spp. via conventional polymerase chain reaction (PCR) targeting the *gltA* gene with BhCS.1137n (5′-AATGCAAAAAGAACAGTAAACA-3′) as forward and BhCS.781p (5′-GGGGaCCaGCTCATGGTGG-3′) as reverse primer (34). All samples were further processed by an additional PCR targeting 453–780 base pairs (bp) of the 16S−23S rRNA intergenic spacer (ITS) region with the forward primer Ba325s (5′-CTTCAGATGATGATCCCAAGCCTTCTGGCG-3′) and the reverse primer Ba1100as (5′-GAACCGACGACCCCCTGCTTGCAAAGCA-3′) (35, 36). Visualization of PCR products followed under UV-light on 2% agarose gel dyed with GelRedTM (Biotium, Hayward CA, USA). As the *gltA* gene is considered to be more sensitive, only samples which were positive in both genes were considered positive and further processed by sequencing. Purification of PCR products of the samples positive in ITS was carried out with the QIAquick PCR purification Kit (Qiagen) according to the manufacturer's recommendations. Purified amplicons were sequenced by Eurofins MWG Operon (Martinsried, Germany) with both primers and sequences were analyzed with Chromas Lite® (Technelysium Pty Ltd, South Brisbane, Australia) as formerly described (35). Obtained sequences were aligned with sequences from GenBank using BLASTn (National Center for Biotechnology Information, Bethesda MD, USA) and deposited in GenBank under following Acc. No.: MT551048-MT551101 and MT913158-MT913206. In total 33% of the positive rodent samples and 29% of the positive flea samples were sequenced. Sequences were considered as a matching result in GenBank with at least a similarity of 97.7%.

### Statistical Analysis

Confidence intervals (95%CI) for the prevalence of *Bartonella* spp. in small mammals and fleas were determined by the Clopper and Pearson method using Graph Pad Software (Graph Pad Software Inc., San Diego, Ca., USA). Independence of compared small sample sizes (*n* < 30) was tested with Fisher's exact test, respectively with the chi-squared test for sample sizes *n* > 30. The *t*-test was used to test significant differences of flea infestation on *My. glareolus* and *A. flavicollis*. The significance threshold was set at *p* ≤ 0.05.

## Results

### Flea Collection

In total, 1,156 fleas were collected from 456 small mammals. Altogether, twelve different flea species were detected (873 *Ctenophthalmus agyrtes*, 3 *Ctenophthalmus bisoctodentatus*, 62 *Ctenophthalmus congener*, 98 *Megabothris turbidus*, 8 *Megabothris walkeri*, 9 *Hystrichopsylla talpae talpae*, 25 *Peromyscopsylla silvatica*, 3 *Paleopsylla soricis*, 44 *Nosopsyllus fasciatus*, 11 *Typhloceras poppei*, 17 *Leptopsylla segnis*) (Table 1). Except for individuals belonging to the insectivore species *T. europaea* and *Sorex* spp., all other small mammal species were infested with fleas. The infestation prevalence ranged from 20 to 100% per small mammal species. The most prevalent species was *C. agyrtes*, which was found on all infested small mammal species. The flea burden was significantly higher on *A. flavicollis* compared to *My. glareolus* (*t* = −91.32; *p* < 0.0001). *Megabothris turbidus* was significantly more often collected from *My. glareolus* than from all other small mammal species (*t* = −5.65; *p* < 0.0001). *Nosopsyllus fasciatus* was significantly more frequently collected from specimens belonging to the family Muridae (*Apodemus* spp.) than from those belonging to the family Cricetidae (*Microtus* spp.; *Myodes* spp.) (*t* = −4.16; *p* = 0.00021). *Leptopsylla segnis* was exclusively found on *A. sylvaticus*, which were trapped only in the urban habitat. *Peromyscopsylla silvatica* were significantly more often collected from *My. glareolus* compared to all other small mammal species (*t* = −3.23; *p* = 0.0006).

**Table 1:** Flea burden per small mammal species.

**Small mammal species**	**Total No.^**1**^ of small mammals**	**Total No.^**1**^ of small mammals infested by fleas (%)**	**Flea species No**.^****1****^ **of small mammals infested/Total No**.^****1****^ **of fleas**											
			***Ctenophthalmus agyrtes***	***Megabothris turbidus***	***Paleopsylla soricis***	***Nosopsyllus fasciatus***	***Ctenophtalmus congener***	***Ctenophthalmus bisoctodentaus***	***Megabothris rectangulatus***	***Typhloceras poppei***	***Hystrichpsylla talpae talpae***	***Leptopsylla segnis***	***Megabothris walkeri***	***Peromyscopsylla silvatica***
*Apodemus flavicollis*	172	146 (84.9)	128/298	12/12	–	15/20	6/9	3/3	1/1	2/3	4/4	–	3/3	1/1
*Myodes glareolus*	395	276 (69.9)	232/507	61/80	2/3	4/4	36/51	-	1/2	1/1	4/4	–	5/5	21/24
*Apodemus agrarius*	6	6 (100)	5/8	1/1-	–	–	1/1	–	–	–	–	–	–	–
*Apodemus sylvaticus*	35	24 (68.6)	16/50	2/2	–	12/20	–	–	–	2/7	1/1	10/17	–	–
*Mustela nivalis*	2	1 (50)	1/4	1/1	–	–	–	–	–	–	–	–	–	–
*Microtus agrestis*	1	1 (100)	1/4	1/2	–	–	–	–	–	–	–	–	–	–
*Microtus arvalis*	6	2 (33.3)	1/2	–	–	–	1/1	–	–	–	–	–	–	–
Total No.^1^	617	456 (73.9)	384/873	78/98	2/3	31/44	43/62	3/3	2/3	5/11	9/9	10/17	8/8	22/25

*No.^1^, Number*.

### *Bartonella* spp. in Rodents

Only samples yielding a positive result in both PCR approaches were considered positive in the following analysis. In total, 43.3% (95%CI: 39.5–47.3) of all small mammals were positive for *Bartonella* spp. Though not infested with fleas, positive *T. europaea* (100%; 95%CI: 16.75–100) and *Sorex* spp. (20%; 95%CI: 0–11.5) were detected. The prevalences were quite high in all captured small mammal species (20–100%) with the exception of *Microtus* spp. which were all negative and thus significantly less often infected than other species (*p* = 0.007). Considering the two most frequently captured species, the prevalence was significantly higher in *A. flavicollis* compared to *My. glareolus* (*p* < 0.0001; Table 2).

**Table 2:** *Bartonella* spp. detection based on the gltA gene and the 16S−23S rRNA ITS and species determination based on the 16S−23S rRNA ITS in spleen samples from different small mammal species.

		**Total No. of small mammals infected with *Bartonella* tested by ITS (%)**	**Total No. of small mammals infected with *Bartonella* based on *gltA* (and ITS) (%)**	**No. of *Bartonella*-positive samples further investigated by sequencing**	**No. of detected** ***Bartonella*** **species based on the 16s-23S rRNA ITS**				
**Small mammal species**	**Total No**.				**Uncultured *Bartonella* sp**.	***Bartonella* sp. N40**	***B. grahamii***	***B. taylorii***	***B. doshiae***
*Myodes glareolus*	395	188 (47.5)	144 (36.5)^***^	44	25	0	7	5	7
*Apodemus flavicollis*	172	129 (73.3)	106 (61.6)^***^	31	21	4	0	5	1
*Apodemus sylvaticus*	35	14 (41.2)	13 (37.1)	12	10	1	0	1	0
*Apodemus agrarius*	6	3 (50)	3 (50)	1	0	0	0	1	0
*Microtus arvalis*	6	0 (0)	0 (0)	–	–	–	–	–	–
*Microtus agrestis*	1	0 (0)	0 (0)	–	–	–	–	–	–
*Mustela nivalis*	2	2 (100)	2 (100)	0	–	–	–	–	–
*Sorex spp*.	5	2 (40)	1 (20)	0	–	–	–	–	–
*Talpa europaea*	1	1 (100)	1 (100)	0	–	–	–	–	–
Total	623	339 (51.4)	270 (43.3)	88	56	5	7	12	8

*** *A. flavicollis significantly higher than in My. glareolus (p = 0.0001)*.

*No., Number; B., Bartonella; sp., species; Cand., Candidatus*.

### Sequence Analysis for *Bartonella* spp. in Rodents

A total of 88 out of 270 (33%) PCR products were selected by small mammal species and location to be further processed in order to determine the *Bartonella* species via sequence analysis. Altogether five different *Bartonella* species were detected in rodents and *Sorex araneus* (Table 2). The most prevalent species group (*n* = 56) which was detected in small mammals were uncultured *Bartonella* species. Altogether 56 sequences obtained in this study showed 100% identity to altogether 16 different uncultured *Bartonella* spp. sequences deposited in GenBank (Table 3), and these sequences showed 27–99% homology to one another. *Bartonella grahamii* was significantly more often detected in *My. glareolus* compared to *A. flavicollis* (*p* = 0.0370). *Bartonella doshiae* was mainly detected in *My. glareolus*. There were four very short sequences (below 430 base pairs) which were not considered as a positive sequencing result and therefore not taken into consideration for Bartonella species identification.

**Table 3:** Number of *Bartonella* sp. sequences based on the 16S−23S rRNA ITS of *Bartonella* found in small mammals and fleas in this study in comparison to sequences from GenBank.

**Host in this study (number of sequences per host species)**	**Number of *Bartonella* sequences detected**	***Bartonella* sp. detected**	**Identity to following Accession number in Genbank**	**Host in GenBank**	**Country of origin**	**Citation of GenBank Accession number**
*Apodemus flavicollis*	2	*Bartonella* sp. uncultured	DQ155391	*Apodemus flavicollis*	Slovenia	(38)
*Ctenophthalmus agyrtes*	3	*Bartonella* sp. uncultured	DQ155391	*Apodemus flavicollis*	Slovenia	(38)
*Apodemus flavicollis* (1); *Myodes glareolus* (1); *Apodemus sylvaticus* (2)	4	*Bartonella* sp. uncultured	DQ155384	*Apodemus sylvaticus*	Slovenia	(38)
*Apodemus flavicollis* (1); *Myodes glareolus* (2);	3	*Bartonella* sp. uncultured	AJ269792	*Apodemus sylvaticus*	UK	(54)
*Ctenophthalmus agyrtes*	1	*Bartonella* sp. uncultured	AJ269794	*Apodemus sylvaticus*	UK	(54)
*Apodemus flavicollis* (2); *Myodes glareolus* (3);	5	*Bartonella* sp. uncultured	DQ155380	*Myodes glareolus*	Slovenia	(38)
*Ctenophthalmus agyrtes* (1); *Peromyscopsylla sylvatica* (1)	2	*Bartonella* sp. uncultured	DQ155380	*Myodes glareolus*	Slovenia	(38)
*Myodes glareolus*	2	*Bartonella* sp. uncultured	DQ155381	*Myodes glareolus*	Slovenia	(38)
*Apodemus flavicollis* (2); *Apodemus sylvaticus* (3)	5	*Bartonella* sp. uncultured	KU886433	*Ctenophthalmus nobilis*	Germany	(24)
*Ctenophthalmus agyrtes*	2	*Bartonella* sp. uncultured	KU886488	*Apodemus flavicollis*	Germany	(24)
*Nosopsyllus fasciatus;*	6	*Bartonella* sp. uncultured	KU886411	*Megabothris turbidus*	Germany	(24)
*Myodes glareolus*	1	*Bartonella* sp. uncultured	KX267701	*Ixodes ricinus*	Slovakia	(55)
*Apodemus flavicollis*	3	*Bartonella* sp. uncultured	MF039571	Rodent	Slovakia	(56)
*Myodes glareolus* (6); *Apodemus flavicollis* (5)	11	*Bartonella* sp. uncultured	MN056366	*Myodes glareolus*	Germany	(43)
*Myodes glareolus*	2	*Bartonella* sp. uncultured	MN056367	*Myodes glareolus*	Germany	(43)
*Myodes glareolus*	1	*Bartonella* sp. Uncultured	MN056369	*Myodes glareolus*	Germany	(43)
*Myodes glareolus* (4); *Apodemus flavicollis* (1)	5	*Bartonella* sp. uncultured	MN056373	*Myodes glareolus*	Germany	(43)
*Myodes glareolus*	2	*Bartonella* sp. uncultured	MN056376	*Myodes glareolus*	Germany	(43)
*Apodemus flavicollis*	4	*Bartonella* sp. uncultured	MN056378	*Apodemus agrarius*	Germany	(43)
*Myodes glareolus*	1	*Bartonella* sp. uncultured	MN056390	*Apodemus sylvaticus*	Germany	(43)
*Apodemus sylvaticus*	5	*Bartonella* sp. uncultured	MN056393	*Microtus arvalis*	Czech Republic	(43)
*Myodes glareolus*	2	*Bartonella taylorii*	MH547342	*Apodemus flavicollis*	Lithuania	(57)
*Myodes glareolus* (3); *Apodemus flavicollis* (5); *Apodemus sylvaticus* (1); *Apodemus agrarius* (1);	10	*Bartonella taylorii*	MH547337	*Apodemus flavicollis*	Lithuania	(57)
*Ctenophthalmus agyrtes* (14) *Peromyscopsylla sylvatica* (1)	15	*Bartonella taylorii*	MH547337	*Apodemus flavicollis*	Lithuania	(57)
*Apodemus flavicollis* (4); *Apodemus sylvaticus* (1);	5	*Bartonella* sp. N40	AJ269787	Genomic DNA	UK	(54)
*Hystrichpsylla talpae talpae* (1) *Ctenophthalmus agyrtes* (4)	5	*Bartonella* sp. N40	AJ269787	Genomic DNA	UK	(54)
*Myodes glareolus*	7	*Bartonella grahamii*	CP001562	Genomic DNA	Sweden	(58)
*Ctenophthalmus agyrtes* (1) *Megabothris turbidus* (1) *Ctenophthalmus congener* (1)	3	*Bartonella grahamii*	CP001562	Genomic DNA	Sweden	(58)
*Hystrichpsylla talpae talpae* (1) *Ctenophthalmus agyrtes* (2)	3	*Bartonella* sp. uncultured	MN056412	*Apodemus flavicollis*	Germany	(43)
*Ctenophthalmus agyrtes*	4	*Bartonella* sp. uncultured	MK562487	*Apodemus* sp.	Italy	(59)
*Leptopsylla segnis* (1) *Ctenophthalmus congener* (2) *Ctenophthalmus agyrtes* (2)	5	*Bartonella* sp. uncultured	MN056379	*Apodemus sylvaticus*	Germany	(43)
*Ctenophthalmus agyrtes*	1	*Bartonella* sp. uncultured	MN056375	*Myodes glareolus*	Germany	(43)
*Ctenophthalmus congener* (1) *Ctenophthalmus agyrtes* (1) Megabothris walkeri (1) *Nosopsyllus fasciatus* (1)	4	*Bartonella taylorii*	MH547339	*Apodemus flavicollis*	Lithuania	(57)
*Ctenophthalmus agyrtes*	11	*Bartonella* sp. uncultured	MT551074	*Apodemus sylvaticus*	Germany	Current study
*Myodes glareolus* (7) *Apodemus flavicollis* (1)	8	*Bartonella doshiae*	AJ269786	genomic DNA	UK	(54)

### *Bartonella* spp. in Fleas

Overall, 221 out of 450 fleas were tested positive for *Bartonella* spp. [49.1% (95%CI: 44.5–53.7)]. Every tested flea species was positive for *Bartonella* spp. with a prevalence ranging from 18.8 to 100% (Table 3). The prevalence levels of *Bartonella* spp. did not differ significantly comparing the most prevalent flea species (*C. agyrtes, M. turbidus, N. fasciatus, C. congener*; χ^2^ = 1.8; *p* = 0.6121). Comparing small mammals and fleas, the prevalence with *Bartonella* spp. was almost identical and thus not significantly different (*p* = 0.9018). Positive fleas derived from 53 negative and 54 positive small mammals.

### Sequence Analysis for *Bartonella* spp. in Fleas

In total, 74 positive samples (29%) were further determined to species level by sequencing which revealed four different *Bartonella* species in the examined fleas. All confirmed *Bartonella* species detected in fleas were the same *Bartonella* species as described for their small mammal hosts. However, most samples were positive for uncultured *Bartonella* spp. which showed 100% identity to 13 different sequences deposited in GenBank (Table 3). Almost all strains found in fleas were identical to those already found in their small mammal hosts (Table 4). Even though the distribution of the *Bartonella* species found in fleas was not completely identical compared to *Bartonella* spp. in small mammals, the prevalence of each *Bartonella* species did not differ significantly between small mammals and fleas (*p* = 0.2418–0.7631). Due to very small sample sizes of most flea species, statistical comparisons between the flea species were not carried out.

**Table 4:** *Bartonella* spp. detection based on the gltA gene and the 16S−23S rRNA ITS and species determination based on the 16S−23S rRNA ITS in fleas collected from small mammals.

**Flea species**	**Total No**.	**No. examined for *Bartonella***	**Total No. of fleas infected with *Bartonella* based on ITS(%)**	**Total No. of fleas infected with *Bartonella* based on gltA (and ITS)(%)**	**No. of *Bartonella*-positive samples further investigated by sequencing**	**No. of detected** ***Bartonella*** **species based on the 16S−23S rRNA ITS**			
						**Uncultured *Bartonella* sp**.	***B. grahamii***	***B. taylorii***	***Bartonella* sp. N40**
*Ctenophthalmus agyrtes*	873	322	213 (59.5)	165 (51.2)	55	35	1	15	4
*Megabothris turbidus*	98	31	15 (48.4)	11(35.5)	3	2	1	0	0
*Nosopsyllus fasciatus*	44	31	18 (58.1)	16 (51.6)	6	5	0	1	0
*Ctenophthalmus congener*	62	23	12 (52.2)	12 (52.2)	3	2	0	1	0
*Megabothris walkeri*	8	3	1 (33.3)	1 (33.3)	1	0	0	1	0
*Typhloceras poppei*	11	2	2 (28.6)	2 (100)	1	0	1	0	0
*Paleopsylla soricis*	3	1	1 (100)	0 (0)	0	–	–	–	–
*Leptopsylla segnis*	17	16	6 (37.5)	3 (18.8)	1	1	0	0	0
*Ctenophthalmus bisoctodentatus*	3	1	1 (100)	1 (100)	0	–	–	–	–
*Hystrichopsylla talpae talpae*	9	3	3 (100)	3 (100)	1	0	0	0	1
*Peromyscopsylla silvatica*	25	18	7 (38.9)	7 (38.9)	3	2	0	1	0
*Megabothris rectangulatus*	3	0	–	–	–	–	–	–	–
Total	1156	450	258 (57.3)	221 (49.1)	74	47	3	19	5

*No., Number*.

## Discussion

This study reports high prevalence rates of different *Bartonella* species in small mammals (43.3%) and their fleas (49.1%) from Germany. The *Bartonella* species detected in the current study were the same as earlier described by our group in small mammals from one of the investigated study sites (urban, renatured, and sylvatic) (24). The prevalences in small mammals from the current study as well as the detected *Bartonella* spp. are in line with those from Poland, France, the Netherlands, Slovenia, and Germany (11–72%) (10, 13, 24, 37, 38). Small mammals are known to be the main reservoirs for over 22 different Bartonella species (7, 8). The current study reports four Bartonella species belonging to lineage four in small mammals (*B. grahamii, B. doshiae, B. taylorii, Bartonella* sp. N40;). Of the detected Bartonella spp., only *B. grahamii* is yet known to be zoonotic. *Bartonella grahamii* was isolated from *My. glareolus* from the UK for the first time (39). Since, it was found in rodents from almost all over the world and also caused disease in humans (40). Bartonellosis caused by *B. grahamii* displays similar symptoms as cat scratch disease such as enlarged lymph nodes, fever and fatigue. Even though cats are not considered competent reservoirs for *B. grahamii*, reports showed that they may still transmit the pathogen to humans via cat scratches when carrying infected rodent tissue on their claws (40). Most cases of bartonellosis caused by *B. grahamii* are likely to remain undiagnosed due to the mild unspecific symptoms, insufficient diagnostic measures and the lack of awareness of practitioners (40). Further, 16 different uncultured *Bartonella* spp. strains of yet unknown pathogenic potential were found in small mammals from the current study. This finding makes it obvious why clinical and public awareness of this zoonotic threat have to be increased.

Regarding the investigated small mammal species, *Apodemus* spp. showed a significantly higher infection rate compared to *My. glareolus* providing evidence that *My. glareolus* is able to resolve *Bartonella* infection after a certain time, while resolving a *Bartonella* infection has not been observed in *Apodemus* spp. yet (10). Moreover, it has been described that the re-infection rate in *Apodemus* spp. is higher than in *My. glareolus* which could also explain the higher prevalence in *Apodemus* spp.

The infestation rate with fleas may also influence the *Bartonella* prevalence in small mammals. In the current study, *Apodemus* spp. were more often infested with fleas and the infestation rate of fleas was higher compared to *My. glareolus*, which was also described in earlier studies from Germany and explained by the larger body size of *Apodemus* spp. (41). This higher infestation rate may have resulted in a higher *Bartonella* prevalence in *Apodemus* spp. High *Bartonella* prevalence rates (36–42%) were reported in *Mi. arvalis* and *Mi. agrestis* from Finland and Poland, respectively (9, 42). Further, another study by our group showed moderate to high prevalence rates in *Microtus* spp. from the Czech Republic and Germany (43). In the current study, both *Microtus* species (*n* = 8) were the only rodent species found negative for *Bartonella* spp. However, the sample size tested was rather low. Although no fleas were found on the insectivores analyzed in this study, all insectivore species were positive for *Bartonella* spp. In Sweden, it has been reported that insectivores may serve as reservoirs for certain *Bartonella* species (37). Future studies need to be conducted in order to confirm this observation. The urban study site was the only site where *A. sylvaticus* were trapped and *L. segnis* were detected. Further *L. segnis* is known to be a vector of *Rickettsia felis* and *Rickettsia typhi* and to occur mainly on small mammals which live synanthropic such as *Mus musculus* and *Rattus norvegicus* (44). The name “*A. sylvaticus*” is misleading as this species is likewise synanthropic and a well-known host for *L. segnis* (45). As the urban study site is a small park surrounded by walls and a high-traffic road, this study suggests it has basically a small ecological niche on its own. The proximity of small mammals to human settlements may pose a risk thus to the health of companion animals and humans.

The flea species may vary in their host specificity of being highly host-specific to being only host-opportunistic (46). The variety of flea species found in this study was high with twelve identified species. This high diversity is quite unexpected as previous studies from Poland, the UK and Germany found only 4–10 different flea species on the mentioned small mammal species (24, 41, 47, 48). However, one should consider that the current study covered three completely differently structured study sites which may have led to a higher variety of flea species. The flea burden was higher on *Apodemus* spp. than on all other small mammal species. It is known that there is a higher immune resistance against flea burden in *Microtus* spp. compared to *Apodemus* spp. and *My. glareolus* (49) which could also explain why the Microtus spp. in the current study were all negative for *Bartonella* spp. In our study, *M. turbidus* and *P. silvatica* were found significantly more often on *My. glareolus* compared to *A. flavicollis* which confirms that *My. glareolus* is the main reservoir host for *M. turbidus* (50). Moreover, the occurrence of *P. silvatica* is quite rare and known to occur on *My. glareolus* suggesting host specificity (51).

The *Bartonella* species detected in small mammals were almost identical compared to those obtained from fleas. However, half of the positive fleas were collected from negative small mammals. This observation indicates that the infection status of fleas can be independent from that of the current small mammal host. A previous study reported the vertical transmission in fleas which could explain how Bartonellae maintain in flea populations independently from a mammalian reservoir (52). Furthermore, frequent host changes by the fleas may have led to high infection levels. Only a few other studies report *Bartonella* prevalence in the examined flea species (15, 17). However, it should be considered that some of these flea species may parasitize companion animals such as cats and dogs (53) and thus may pose a health threat as direct vectors of zoonotic pathogens such as *Bartonella* spp. and *Rickettsia* spp.

To conclude, this study shows a high diversity of flea species on small mammal hosts from Germany. Though none of the detected vector-host species combinations was unusual, the number of flea species found was unexpectedly high. In addition to small mammals, some of them especially the ones collected at the urban site also parasitize companion animals such as dogs and cats and may pose a risk for the transmission of zoonotic *Bartonella* spp. Though *B. grahamii* was the only confirmed zoonotic *Bartonella* in this study, a very high variety of uncultured *Bartonella* spp. of yet unknown zoonotic potential was also detected. Especially at the urban study site, a health risk in encountering *Bartonella* infections is possible as infested rodents live there in close proximity to human settlements.

## Data Availability Statement

The datasets presented in this study can be found in online repositories. The names of the repository/repositories and accession number(s) can be found at: https://www.ncbi.nlm.nih.gov/genbank/, MT551048-MT551101; MT913158-MT913206.

## Author Contributions

CS and MP organized and planned the study. AO and MP organized and participated in the fieldwork for the collection of wildlife samples. MK and DK carried out the morphologic determination of fleas. AO prepared the samples in the laboratory. AO and NK tested the samples for *Bartonella* spp. AO, NK, and CS performed the sequence analysis. AO, NK, CS, and MP drafted the manuscript and wrote the final version. All authors read and approved the final manuscript.

## Conflict of Interest

The authors declare that the research was conducted in the absence of any commercial or financial relationships that could be construed as a potential conflict of interest.

## References

[1] Prutsky G Domecq JP Mori L Bebko S Matzumura M Sabouni A. Treatment outcomes of human bartonellosis: a systematic review and meta-analysis. Int J Infect Dis. (2013) 17:e811-9. 10.1016/j.ijid.2013.02.016 23602630

[2] Battisti JM Lawyer PG Minnick MF. Colonization of *Lutzomyia verrucarum* and *Lutzomyia longipalpis* Sand Flies (Diptera: Psychodidae) by *Bartonella bacilliformis*, the Etiologic Agent of Carrión's Disease. PLoS Negl Trop Dis. (2015) 9:e0004128. 10.1371/journal.pntd.0004128 26436553 PMC4593541

[3] Bonilla DL Kabeya H Henn J Kramer VL Kosoy MY. *Bartonella quintana* in body lice and head lice from homeless persons, San Francisco, California, USA. Emerg Infect Dis. (2009) 15:912–5. 10.3201/eid1506.090054 19523290 PMC2727331

[4] Dehio C Sauder U Hiestand R. Isolation of *Bartonella schoenbuchensis* from *Lipoptena cervi*, a blood-sucking arthropod causing deer ked dermatitis. J Clin Microbiol. (2004) 42:5320–3. 10.1128/JCM.42.11.5320-5323.2004 15528732 PMC525279

[5] Rolain J-M Franc M Davoust B Raoult D. Molecular detection of *Bartonella quintana, B. koehlerae, B. henselae, B. clarridgeiae, Rickettsia felis*, and *Wolbachia pipientis* in cat fleas, France. Emerg Infect Dis. (2003) 9:339–42. 10.3201/eid0903.020278 12643829 PMC2958535

[6] Harms A Dehio C. Intruders below the radar: molecular pathogenesis of *Bartonella* spp. Clin Microbiol Rev. (2012) 25:42–78. 10.1128/CMR.05009-11 22232371 PMC3255967

[7] Deng H Le Rhun D Buffet J-PR Cotté V Read A Birtles RJ. Strategies of exploitation of mammalian reservoirs by Bartonella species. Vet Res. (2012) 43:15. 10.1186/1297-9716-43-15 22369683 PMC3430587

[8] Wagner A Dehio C. Role of distinct type-IV-secretion systems and secreted effector sets in host adaptation by pathogenic Bartonella species. Cell Microbiol. (2019) 21:e13004. 10.1111/cmi.13004 30644157 PMC6519360

[9] Gutiérrez R Krasnov B Morick D Gottlieb Y Khokhlova IS Harrus S. Bartonella infection in rodents and their flea ectoparasites: an overview. Vector Borne Zoonotic Dis. (2015) 15:27–39. 10.1089/vbz.2014.1606 25629778 PMC4307031

[10] Paziewska A Harris PD Zwolińska L Bajer A Siński E. Differences in the ecology of Bartonella infections of *Apodemus flavicollis* and *Myodes glareolus* in a boreal forest. Parasitology. (2012) 139:881–93. 10.1017/S0031182012000170 22336264

[11] Welc-Faleciak R Bajer A Behnke JM Siński E. The ecology of *Bartonella* spp. infections in two rodent communities in the Mazury Lake District region of Poland. Parasitology. (2010) 137:1069–77. 10.1017/S0031182009992058 20388232

[12] Andersson M Råberg L. Wild rodents and novel human pathogen candidatus *Neoehrlichia mikurensis*, Southern Sweden. Emerg Infect Dis. (2011) 17:1716–8. 10.3201/eid1709.101058 21888802 PMC3322053

[13] Buffet J-P Marsot M Vaumourin E Gasqui P Masséglia S Marcheteau E. Co-infection of *Borrelia afzelii* and *Bartonella* spp. in bank voles from a suburban forest. Compar Immunol Microbiol Infect Dis. (2012) 35:583–9. 10.1016/j.cimid.2012.07.002 22898354

[14] Telfer S Clough HE Birtles RJ Bennett M Carslake D Helyar S. Ecological differences and coexistence in a guild of microparasites: Bartonella in wild rodents. Ecology. (2007) 88:1841–9. 10.1890/06-1004.1 17645030

[15] Billeter SA Levy MG Chomel BB Breitschwerdt EB. Vector transmission of Bartonella species with emphasis on the potential for tick transmission. Med Vet Entomol. (2008) 22:1–15. 10.1111/j.1365-2915.2008.00713.x 18380649

[16] Tsai Y-L Chang C-C Chuang S-T Chomel BB Bartonella species and their ectoparasites: selective host adaptation or strain selection between the vector and the mammalian host? Compar Immunol Microbiol Infect Dis. (2011) 34:299–314. 10.1016/j.cimid.2011.04.005 21616536

[17] Bown KJ Bennet M Begon M. Flea-borne *Bartonella grahamii* and *Bartonella taylorii* in bank voles. Emerg Infect Dis. (2004) 10:684–7. 10.3201/eid1004.030455 15200860 PMC3323072

[18] Morick D Krasnov BR Khokhlova IS Gutiérrez R Gottlieb Y Harrus S. Vertical nontransovarial transmission of Bartonella in fleas. Mol Ecol. (2013) 22:4747–52. 10.1111/mec.12408 23875817

[19] Reeves WK Rogers TE Durden LA Dasch GA. Association of Bartonella with the fleas (Siphonaptera) of rodents and bats using molecular techniques. J Vector Ecol. (2007) 32:118–22. 10.3376/1081-1710(2007)32[118:AOBWTF]2.0.CO;2 17633432

[20] Morick D Krasnov BR Khokhlova IS Shenbrot GI Kosoy MY Harrus S. Bartonella genotypes in fleas (insecta: siphonaptera) collected from rodents in the negev desert, Israel. Appl Environ Microbiol. (2010) 76:6864–9. 10.1128/AEM.00879-10 20802081 PMC2953035

[21] Billeter SA Gundi VA Rood MP Kosoy MY. Molecular detection and identification of Bartonella species in *Xenopsylla cheopis* fleas (Siphonaptera: Pulicidae) collected from *Rattus norvegicus* rats in Los Angeles, California. Appl Environ Microbiol. (2011) 77:7850–2. 10.1128/AEM.06012-11 21908631 PMC3209143

[22] Marié J-L Fournier P-E Rolain J-M Briolant S Davoust B Didier R. Molecular detection of *Bartonella quintana, B. Elizabethae, B. Koehlerae, B. Doshiae, B. Taylorii*, and *Rickettsia felis* in rodent fleas collected in Kabul, Afghanistan. Am J Trop Med Hyg. (2006) 74:436–9. 10.4269/ajtmh.2006.74.436 16525103

[23] Lewis RE. Notes on the geographical distribution and host preferences in the order Siphonaptera. 1. Pulicidae. J Med Entomol. (1972) 9:511–20. 10.1093/jmedent/9.6.511 4654683

[24] Silaghi C Pfeffer M Kiefer D Kiefer M Obiegala A. Bartonella, rodents, fleas and ticks: a molecular field study on host-vector-pathogen associations in Saxony, Eastern Germany. Microb Ecol. (2016) 72:965–74. 10.1007/s00248-016-0787-8 27220973

[25] Silaghi C Woll D Hamel D Pfister K Mahling M Pfeffer M. *Babesia* spp. and *Anaplasma phagocytophilum* in questing ticks, ticks parasitizing rodents and the parasitized rodents–analyzing the host-pathogen-vector interface in a metropolitan area. Parasit Vectors. (2012) 5:191. 10.1186/1756-3305-5-191 22950642 PMC3480827

[26] Obiegala A Pfeffer M Pfister K Tiedemann T Thiel C Balling A. Candidatus *Neoehrlichia mikurensis* and *Anaplasma phagocytophilum*: prevalences and investigations on a new transmission path in small mammals and ixodid ticks. Parasit Vectors. (2014) 7:563. 10.1186/s13071-014-0563-x 25465390 PMC4264555

[27] Overzier E Pfister K Herb I Mahling M Böck G Silaghi C. Detection of tick-borne pathogens in roe deer (*Capreolus capreolus*), in questing ticks (*Ixodes ricinus*), and in ticks infesting roe deer in southern Germany. Ticks Tick Borne Dis. (2013) 4:320–8. 10.1016/j.ttbdis.2013.01.004 23571115

[28] Overzier E Pfister K Thiel C Herb I Mahling M Silaghi C. Diversity of Babesia and Rickettsia species in questing *Ixodes ricinus*: a longitudinal study in urban, pasture, and natural habitats. Vector Borne Zoonotic Dis. (2013) 13:559–64. 10.1089/vbz.2012.1278 23697771 PMC3741418

[29] Angermann R Senglaub K Stresemann E (Editors). Wirbeltiere. Heidelberg: Spektrum Akademischer Verlag (2011).

[30] Parson W K. Pegoraro K Niederstätter H Föger M Steinlechner M. Species identification by means of the cytochrome b gene. Int J Leg Med. (2000) 114:23–8. 10.1007/s004140000134 11197623

[31] Peus F (Editor). Zur Kenntnis der Flöhe Deutschlands. Faunistik und Ökologie der Säugetierflöhe. Insectivora, Lagomorpha, Rodentia. Jena: Zoologische Jahrbücher für Systematik (1970).

[32] Peus F (Editor). Zur Kenntnis Flöhe Deutschlands. – IV. Faunistik und Ökologie der Säugetierflöhe. Jena: Zoologische Jahrbücher für Systematik (1972).

[33] Obiegala A Pfeffer M Pfister K Karnath C Silaghi C. Molecular examinations of *Babesia microti* in rodents and rodent-attached ticks from urban and sylvatic habitats in Germany. Ticks Tick Borne Dis. (2015) 6:445–9. 10.1016/j.ttbdis.2015.03.005 25922232

[34] Norman AF Regnery R Jameson P Greene C Krause DC. Differentiation of Bartonella-like isolates at the species level by PCR-restriction fragment length polymorphism in the citrate synthase gene. J Clin Microbiol. (1995) 33:1797–803. 7545181 10.1128/jcm.33.7.1797-1803.1995 PMC228273

[35] Maggi RG Diniz PP Cadenas MB Breitschwerdt EB (Editor). The use of molecular diagnostic techniques to detect Anaplasma, Bartonella and Ehrlichia species in arthropods or patients. The International Canine Vector-Borne Disease Symposium. Billesley: Alcester (2006).

[36] Schorn S Pfister K Reulen H Mahling M Silaghi C. Occurrence of Babesia spp., Rickettsia spp. and Bartonella spp. in Ixodes ricinus in Bavarian public parks, Germany. Parasit Vectors (2011) 4:135. 10.1186/1756-3305-4-135 21762494 PMC3154157

[37] Holmberg M Mills JN McGill S Benjamin G Ellis BA. Bartonella infection in sylvatic small mammals of central Sweden. Epidemiol Infect. (2003) 130:149–57. 10.1017/s0950268802008075 12613756 PMC2869949

[38] Knap N Duh D Birtles R Trilar T Petrovec M Avsic-Zupanc T. Molecular detection of Bartonella species infecting rodents in Slovenia. FEMS Immunol Med Microbiol. (2007) 50:45–50. 10.1111/j.1574-695X.2007.00226.x 17374132

[39] Birtles RJ Harrison TG Saunders NA Molyneux DH. Proposals to unify the genera Grahamella and Bartonella, with descriptions of *Bartonella talpae* comb. nov., *Bartonella peromysci* comb. nov., and three new species, *Bartonella grahamii* sp. nov., *Bartonella taylorii* sp. nov., and *Bartonella doshiae* sp. nov. Int J Syst Bacteriol. (1995) 45:1–8. 10.1099/00207713-45-1-1 7857789

[40] Oksi J Rantala S Kilpinen S Silvennoinen R Vornanen M Veikkolainen V. Cat scratch disease caused by *Bartonella grahamii* in an immunocompromised patient. J Clin Microbiol. (2013) 51:2781–4. 10.1128/JCM.00910-13 23740723 PMC3719609

[41] Maaz D Krücken J Blümke J Richter D McKay-Demeler J Matuschka F-R. Factors associated with diversity, quantity and zoonotic potential of ectoparasites on urban mice and voles. PLoS ONE. (2018) 13:e0199385. 10.1371/journal.pone.0199385 29940047 PMC6016914

[42] Huitu O Aaltonen K Henttonen H Hirvelä-Koski V Forbes K Perez-Vera C Field voles Microtus agrestis as reservoirs of Bartonella spp. EDENext. Montpellier (2015).

[43] Obiegala A Jeske K Augustin M Król N Fischer S Mertens-Scholz K. Highly prevalent bartonellae and other vector-borne pathogens in small mammal species from the Czech Republic and Germany. Parasit Vectors. (2019) 12:1–8. 10.1186/s13071-019-3576-7 31269975 PMC6610854

[44] Christou C Psaroulaki A Antoniou M Toumazos P Ioannou I Mazeris A. *Rickettsia typhi* and *Rickettsia felis* in *Xenopsylla cheopis* and *Leptopsylla segnis* Parasitizing Rats in Cyprus. Am J Trop Med Hyg. (2010) 83:1301–4. 10.4269/ajtmh.2010.10-0118 21118938 PMC2990048

[45] Fairley JS Fleas from the Fieldmouse *Apodemus sylvaticus* (L.) in Co. Down. Irish Nat J. (1963) 14:145–9.

[46] Krasnov BR Shenbrot GI Khokhlova IS Allan Degen A Relationship between host diversity and parasite diversity: flea assemblages on small mammals. J Biogeogr. (2004) 31:1857–66. 10.1111/j.1365-2699.2004.01132.x

[47] Noyes HA Ambrose P Barker F Begon M Bennet M Bown KJ Host specificity of Trypanosoma (Herpetosoma) species: evidence that bank voles (*Myodes glareolus*) carry only one T. (H.) evotomys 18S rRNA genotype but wood mice (*Apodemus sylvaticus*) carry at least two polyphyletic parasites. Parasitology. (2002) 124:185–90. 10.1017/s0031182001001019 11860034

[48] Karbowiak G Solarz K Asman M Wróblewski Z Slivinska K Werszko J Phoresy of astigmatic mites on ticks and fleas in Poland. Biol. Lett. (2013) 50:89–96. 10.2478/biolet-2013-0007

[49] Krasnov BR Stanko M Miklisova D Morand S. Habitat variation in species composition of flea assemblages on small mammals in central Europe. Ecol Res. (2006) 21:460–9. 10.1007/s11284-005-0142-x21392290

[50] Vashchenok VS Species composition, abundance, and annual cycles of fleas (Siphonaptera) on bank voles (*Myodes glareolus*) in the western part of Vologda Province (Babaevo District). Entmol Rev. (2014) 94:359–66. 10.1134/S0013873814030087 25464742

[51] Fairley JS Epifauna from Irish bank voles *Myodes glareolus* schreber. Irish Nat J. (1970) 16:342–6.

[52] Brinkerhoff RJ Kabeya H Inoue K Bai Y Maruyama S. Detection of multiple Bartonella species in digestive and reproductive tissues of fleas collected from sympatric mammals. ISME J. (2010) 4:955–8. 10.1038/ismej.2010.22 20220787

[53] Visser M Rehbein S Wiedemann C. Species of flea (siphonaptera) infesting pets and hedgehogs in Germany. J Vet Med B Infect Dis Vet Public Health. (2001) 48:197–202. 10.1046/j.1439-0450.2001.00445.x 11393815

[54] Birtles RJ Hazel S Bown K Raoult D Begon M Bennett M Subtyping of uncultured bartonellae using sequence comparison of 16 S/23 S rRNA intergenic spacer regions amplified directly from infected blood. Mol Cell Probes. (2000) 14:79–87. 10.1006/mcpr.2000.0289 10799268

[55] Kraljik J Paziewska-Harris A Miklisová D Blanarová L Mošanský L Bona M. Genetic diversity of Bartonella genotypes found in the striped field mouse (*Apodemus agrarius*) in Central Europe. Parasitology. (2016) 143:1437–42. 10.1017/S0031182016000962 27279125

[56] Špitalská E Minichová L Kocianová E Škultéty L Mahríková L Hamšíková Z. Diversity and prevalence of Bartonella species in small mammals from Slovakia, Central Europe. Parasitol Res. (2017) 116:3087–95. 10.1007/s00436-017-5620-x 28975409

[57] Mardosaite-Busaitiene D Radzijevskaja J Balčiauskas L Bratchikov M Jurgelevičius V Paulauskas A. Prevalence and diversity of Bartonella species in small rodents from coastal and continental areas. Sci Rep. (2019) 9:12349. 10.1038/s41598-019-48715-y 31451710 PMC6710269

[58] Berglund EC Frank AC Calteau A Vinnere Pettersson O Granberg F Eriksson A-S. Run-off replication of host-adaptability genes is associated with gene transfer agents in the genome of mouse-infecting *Bartonella grahamii*. PLoS Genet. (2009) 5:e1000546. 10.1371/journal.pgen.1000546 19578403 PMC2697382

[59] Divari S Pregel P Zanet S Ferroglio E Giannini F Scaglione FE PCR detection of Bartonella spp. in rats (*Rattus rattus*) and mice (*Apodemus* spp.) of Pianosa Island, Italy. J Compar Pathol. (2019) 166:141 10.1016/j.jcpa.2018.10.131

